# Guideline of transthyretin-related hereditary amyloidosis for clinicians

**DOI:** 10.1186/1750-1172-8-31

**Published:** 2013-02-20

**Authors:** Yukio Ando, Teresa Coelho, John L Berk, Márcia Waddington Cruz, Bo-Göran Ericzon, Shu-ichi Ikeda, W David Lewis, Laura Obici, Violaine Planté-Bordeneuve, Claudio Rapezzi, Gerard Said, Fabrizio Salvi

**Affiliations:** 1 Department of Neurology, Graduate School of Medical Sciences, Kumamoto University, 1-1-1 Honjo , Chuo-ku, Kumamoto, 860-8556, Japan; 2 Department of Neurology, Hospital de Santo António, Porto, Portugal; 3 Amyloid Treatment & Research Program, Department of Medicine, Boston University, Boston, Massachusetts, USA; 4 Department of Neurology, Federal University of Rio de Janeiro, Hospital Universitário Clementino Fraga Filho, Rio de Janeiro, Brazil; 5 Division of Transplantation Surgery, Karolinska University Hospital, Stockholm, Sweden; 6 Department of Medicine, Shinshu University, Matsumoto, Japan; 7 Department of Transplantation, Lahey Clinic Medical Center, Burlington, Massachusetts, USA; 8 Amyloidosis Research and Treatment Center, Fondazione IRCCS Policlinico San Matteo, Pavia, Italy; 9 Department of Neurology, CHU Henri Mondor, Créteil, France; 10 Institute of Cardiology, University of Bologna, Bologna, Italy; 11 Department of Neurology, Hôpital de la Salpêtrière, Paris, France; 12 Department of Neurology, Istituto delle Scienze Neurologiche di Bologna, Bologna, Italy; 13 Department of Neurology, Graduate School of Medical Sciences, Kumamoto University, 1-1-1 Honjo, Kumamoto, 860-0811, Japan

**Keywords:** Amyloidosis, Polyneuropathy, Cardiomyopathy, Oculoleptomeningeal, Transthyretin, Liver transplant, Genetics

## Abstract

Transthyretin amyloidosis is a progressive and eventually fatal disease primarily characterized by sensory, motor, and autonomic neuropathy and/or cardiomyopathy. Given its phenotypic unpredictability and variability, transthyretin amyloidosis can be difficult to recognize and manage. Misdiagnosis is common, and patients may wait several years before accurate diagnosis, risking additional significant irreversible deterioration. This article aims to help physicians better understand transthyretin amyloidosis—and, specifically, familial amyloidotic polyneuropathy—so they can recognize and manage the disease more easily and discuss it with their patients. We provide guidance on making a definitive diagnosis, explain methods for disease staging and evaluation of disease progression, and discuss symptom mitigation and treatment strategies, including liver transplant and several pharmacotherapies that have shown promise in clinical trials.

## Introduction

Transthyretin (TTR) amyloidosis is a systemic disorder characterized by the extracellular deposition of amyloid fibrils composed of TTR, a plasma transport protein for thyroxine and vitamin A that is produced predominantly by the liver. TTR can dissociate from its native tetramer form, then misfold and aggregate into amyloid fibrils that accumulate in various organs and tissues, causing progressive dysfunction. TTR amyloidosis is the most common form of hereditary (familial) amyloidosis, and is caused by mutations that destabilize the TTR protein. TTR amyloidosis also encompasses an age-related amyloidosis known as senile systemic amyloidosis, an acquired disorder mainly affecting men after the age of 60 years, that results from the deposition of wild-type TTR amyloid.

TTR amyloidosis can present as a progressive, axonal sensory autonomic and motor neuropathy (familial amyloidotic polyneuropathy; TTR-FAP, also known as FAP or ATTR-PN) or as an infiltrative cardiomyopathy (familial amyloid cardiomyopathy). TTR amyloidosis, including TTR-FAP, presents in many different forms, with considerable phenotypic variation across individuals and geographic locations. Diagnosis can be challenging and treatment often requires a multidisciplinary approach. Physicians likely to diagnose and treat patients with this disease include neurologists, cardiologists, gastroenterologists, ophthalmologists, and other specialists.

The recommendations offered here, which focus primarily on the management of polyneuropathy symptoms, are based on the published literature, information gleaned from the Transthyretin Amyloidosis Outcomes Survey (THAOS) – a TTR amyloidosis patient registry, and the opinions of the authors. To further assist the treating physician, Appendix A contains lists of recommended reading and helpful websites.

## Background

In 1952 Andrade reported a large group of patients in Portugal who had TTR-FAP with the Val30Met *TTR* mutation [1]. Over the next two decades other large foci were discovered in Japan and Sweden. Initially, TTR-FAP was thought to be restricted to endemic occurrences in those areas. However, owing to progress in biochemical and molecular genetic analyses, TTR-FAP is now diagnosed worldwide. Most cases involve small kindreds or patients with no family history of the disease. To date, about 120 different single or double mutations, or a deletion in the *TTR* gene, have been reported; the majority of these *TTR* mutations are amyloidogenic, with fewer than ten considered non-pathogenic [2,3]. Val30Met is the most common mutation and the only one found in large foci of patients.

Some mutations induce cardiomyopathy as the predominant feature (e.g. Val122Ile, Ile68Leu, Thr60Ala, Leu111Met) while others are associated primarily with neuropathy (e.g. Val30Met), but both manifestations can be present in different proportions [4]. Less common disease signs include vitreous opacities, renal disease, and meningeal involvement. While genotype and population origin are important determinants of symptoms, the clinical picture of an affected individual may deteriorate over time (as amyloid continues to deposit in other tissues), and variability may be observed within the same family.

### Genetics

TTR-FAP has autosomal dominant inheritance with variable penetrance. Carriers of the mutation have a circulating variant protein from fetal life but no amyloid deposition or symptomatic disease until adulthood, with development of disease probably controlled by factors associated with the biochemistry of aging [3,5]. The penetrance of the gene varies in different regions of the world and among families [6-8]. There is some evidence that affected women transmit higher disease penetrance to their offspring than affected men [9]. Because penetrance is incomplete, carriers of the gene may live to an advanced age without symptoms of the disease but may see their children become clinically affected. Genetic anticipation (earlier onset with greater severity in subsequent generations) has been observed in endemic regions [6,10-12].

### Prevalence and age at onset of TTR amyloidosis

Val30Met is the most prevalent TTR-FAP mutation in the world, focused in Portugal, Sweden, Japan, Brazil, and Majorca, and is believed to have arisen independently in Portugal and Sweden [13-15]. The largest cluster of individuals with TTR-FAP caused by the Val30Met mutation may be found in northern Portugal (Póvoa de Varzim and Vila do Conde), where the incidence is estimated to be one in 538 individuals [16]. In contrast, the incidence of hereditary TTR amyloidosis in the United States is estimated to be one in 100,000 individuals [17]. The cardiomyopathy-related Leu111Met and Val122Ile mutations are found primarily in Danish and African American populations, respectively. However, all mutations, including Val30Met, are identified across all countries in different families without any obvious relationship.

In Europe, the prevalence of TTR-FAP is estimated to be less than one in 100,000 individuals [18]. In endemic areas of northern Sweden (Piteå and Skelleftå), the frequency of the Val30Met mutation is 4%; however, the penetrance is relatively low (11% by 50 years) [6]. Conversely, in Portugal, the penetrance is high (80% by 50 years) [7]. Although also endemic in some areas of Japan, the prevalence of TTR-FAP is estimated to be lower than in Europe, at approximately one in 1,000,000 individuals [19].

The frequency of the Val122Ile mutation in the African American population is 3% to 3.9% [20,21], with most individuals developing late-onset cardiac amyloidosis. The frequency of Val122Ile in Caucasian and Hispanic populations in the United States is 0.44% and 0%, respectively [21]. The worldwide prevalence of TTR amyloidosis dominated by cardiomyopathy is unknown, but it is almost certainly underdiagnosed, particularly in the African American Val122Ile carrier population older than 65 years (approximately 135,000 individuals) [22,23].

The age at onset of disease-related symptoms varies between the second and ninth decades of life, with great variations across different populations. Expected age at onset is critical to determine when amyloidosis testing should be requisitioned. Portuguese and Japanese foci of patients with TTR-FAP have traditionally been described as early-onset (mean age, 33 years) [24,25], whereas Swedish patients with TTR-FAP are characterized by a later mean age of onset (56 years) [26]. Even in foci generally considered early-onset, some subgroups of patients experience a later onset. In Japan, especially, a form of later-onset TTR-FAP with no genealogical relationship to the two foci of this disease now predominates [24,27]. Patients with cardiomyopathy who have wild-type or variant *TTR* traditionally develop symptoms in their sixties.

The mean duration of disease onset to death is approximately 10 years but may vary depending on endemic region, genotype, symptoms, and other factors.

### Clinical presentation of TTR amyloidosis with polyneuropathy (TTR-FAP)

TTR-FAP is a multi-symptom disease that may present with peripheral neuropathy (sensory and motor), autonomic neuropathy, gastrointestinal impairment, cardiomyopathy, nephropathy, or ocular deposition. While the symptoms described below, including those of Val30Met TTR-FAP patients, may be present in patients with different TTR-FAP genotypes, phenotypes are not always uniform, and the same point mutation may have varied phenotypes even within the same family. Generally, however, most TTR-FAP cases are classified as neuropathic.

#### Variation in neuropathies of Val30Met TTR-FAP

The clinical picture for Val30Met TTR-FAP differs considerably between patients originating from endemic foci and patients with non-endemic origins [28]. In the former, disease onset often occurs before the age of 40 years, with a progressive sensory-motor and autonomic neuropathy, eventually causing cachexia and death 10 to 20 years after onset. Although sensory and motor manifestations are generally the presenting symptoms, the first clinical presentation can be autonomic neuropathy. However, even when the disease starts with sensory neuropathy, autonomic neuropathies often follow [29]. Patients with Val30Met TTR-FAP and non-endemic origins typically have onset at a late age (usually after the age of 60), with male predominance and an apparently sporadic disease presentation. Sensory and motor neuropathy symptoms of both upper and lower extremities may appear within a short period or even simultaneously, while autonomic neuropathies may be relatively mild [30-32].

#### Peripheral nerve dysfunction

TTR amyloidosis induces a length-dependent peripheral neuropathy. Initially the lower limbs are affected, and symptoms generally include toe discomfort such as numbness and spontaneous pain. At this stage, as amyloid typically first affects small nerve fibers altering pain and temperature sensation, clinical examination may detect impaired thermal sensitivity in the feet, with decreased pinprick sensation. In contrast, light touch may be relatively preserved and proprioception spared. Muscle strength and tendon reflexes are normal. This neuropathic manifestation typically reflects the involvement of unmyelinated and small myelinated fibers. A few months later sensory loss extends above the ankle on both sides. The neurologic deficit then progresses relentlessly, with the extension of sensory loss toward the proximal lower limbs. Motor deficit appears in the distal lower limbs, as does the impairment of light touch and deep sensations, which indicates the involvement of larger sensory and motor nerve fibers. Walking becomes increasingly difficult with loss of balance and steppage gait. Neuropathic pain is often of the burning type and is worse at night and associated with allodynia. As months and years pass, sensory deficit extends to the thighs, then the upper limbs, forearms, and fingers as the anterior trunk is involved. Motor deficits also follow a length-dependent progression and walking without assistance becomes increasingly difficult. Life-threatening autonomic dysfunction is generally present at this stage along with weight loss and muscle wasting. Loss of pain sensation with preservation of strength leads to painless trauma and the development of plantar ulcers and foot osteoarthropathy (Charcot’s joints) [33]. Due to the random distribution of amyloid in the peripheral nervous system, deposits may accumulate locally and induce focal deficit of a cranial nerve, nerve trunk, or plexus. Carpal tunnel syndrome is an early but nonspecific manifestation of TTR-FAP. It should be noted that many patients with TTR-FAP are erroneously diagnosed with simple carpal tunnel syndrome; progressive symptoms or lack of improvement after carpal tunnel release surgery often leads to the correct diagnosis.

In patients who have peripheral neuropathy of unknown origin, testing for autonomic dysfunction should be considered because patients may not show overt symptoms of autonomic failure. Early recognition of autonomic failure may lead to an earlier diagnosis of the underlying pathogenesis of amyloidosis [34].

#### Other system involvement

Autonomic nervous system involvement includes anhidrosis, sexual impotence, disturbances of gastrointestinal motility (most commonly diarrhea alternating with constipation, but also constipation, diarrhea, nausea, and vomiting), orthostatic hypotension, and neurogenic bladder. In our experience, cardiac disease occurs in approximately 50% of patients with TTR-FAP, with most *TTR* mutations causing amyloid cardiomyopathy [35]. Anemia due to low erythropoietin levels also may be observed [36]. Ocular involvement, such as vitreous opacity, dry eye, glaucoma, and pupillary disorders, is common [37]. In contrast, kidney involvement is unusual.

Additional symptoms of TTR-FAP include hoarseness, coldness, decreased skin temperature, dyscoria, dysesthesia, muscle weakness and atrophy, dissociated anesthesia, and constitutional conditions such as weight loss, arrhythmia, edema, and burning.

#### When should a neurologist suspect TTR-FAP?

It is important to differentiate a diagnostic process in individuals with a known amyloid family history, especially in endemic areas, from that in patients without such a history (Table 1). In patients with a known family history of TTR-FAP, the onset of symptoms and signs of peripheral neuropathy, manifestations of autonomic dysfunction, and cardiac arrhythmia call for confirmation of the involvement of these organs by appropriate investigations (see Tests and Assessments, below). When there is no known family history of amyloidosis, the diagnosis of TTR-FAP should be considered in patients with a progressive, length-dependent axonal polyneuropathy predominantly affecting temperature and pain sensation. Special attention must be paid to patients with concurrent autonomic dysfunction, cardiac involvement, and carpal tunnel syndrome.

**Table 1:** Diagnostic tests for transthyretin (TTR) amyloidosis

**Method**	**Materials**	**Sensitivity**	**Specificity**	**Throughput**	**Cost**	**Aim of Analysis**
*Pathologic*						
Congo red	Tissues	Medium-high	High	High	Low	Detecting amyloid deposits
BSB, FSB dyes	Tissues	High	Medium	High	Low-medium	Detecting amyloid deposits
Electron microscopy	Tissues	Medium	High	Low	Low	Confirming amyloid fibrils
Immunohistochemistry with anti-TTR antibodies	Tissues	High	Medium-high	Low-medium	Low-medium	Detecting TTR deposits
*Genetic*						
PCR-RFLP	DNA	High	High	Medium	Low	Detecting predicted mutations in the *TTR* gene
Real-time PCR (melting curve analysis)	DNA	High	High	High	Medium	Detecting predicted mutations in the *TTR* gene
PCR-SSCP	DNA	Medium	Medium	Medium-high	Low	Screening for unknown mutations in the *TTR* gene
Sequencing	DNA	High	High	Low	High	Detecting unknown mutations in the *TTR* gene
*Mass Spectrometry (MS)*						
MALDI-TOF MS, ESI-MS	Serum protein	Medium-high	Medium	Medium	Low	Detecting variant TTR
FT-ICR MS	Serum protein	Medium-high	Medium-high	Medium	Low	Detecting variant TTR
SELDI-TOF MS	Serum protein	Medium-high	Medium	High	Low-medium	Detecting variant TTR
LC-MS/MS	Tissues	Medium	Medium	Low	Medium	Identifying precursor proteins of amyloid fibrils, including variant TTR

Abbreviations: BSB, 1-Bromo-2,5-bis(3-carboxy-4-hydroxystyryl)benzene; FSB, 1-Fluoro-2,5-bis(3-carboxy-4-hydroxystyryl)benzene; PCR, polymerase chain reaction; RFLP, restriction fragment length polymorphism; SSCP, single-strand conformation polymorphism; MS, mass spectrometry; MALDI-TOF, matrix-assisted laser desorption/ionization time-of-flight; ESI, electrospray ionization; FT-ICR, Fourier transform ion cyclotron resonance; SELDI-TOF, surface enhanced laser desorption/ionization-TOF; LC-MS/MS, liquid chromatography–tandem mass spectrometry.

The multisystem involvement of TTR-FAP is a clue to the diagnosis. It is important to consider the diagnosis of TTR-FAP when one or, especially, several of the following are present:

• Family history of neuropathic disease, especially associated with heart failure

• Neuropathic pain or progressive sensory disturbances of unknown etiology

• Carpal tunnel syndrome without obvious cause, particularly if it is bilateral and requires surgical release

• Gastrointestinal motility disturbances or autonomic nerve dysfunction of unknown etiology (e.g. erectile dysfunction, orthostatic hypotension, neurogenic bladder)

• Cardiac disease characterized by thickened ventricular walls in the absence of hypertension

• Advanced atrio-ventricular block of unknown origin, particularly when accompanied by a thickened heart

• Vitreous body inclusions of the cotton-wool type

### TTR amyloidosis with cardiomyopathy

Although TTR amyloidosis is considered principally a neurologic disease, the clinical spectrum varies widely from almost exclusive neurologic involvement within an endemic cohort to a strictly cardiologic presentation in sporadic cases [38-41]. TTR-related cardiac amyloidosis is thought to be vastly underdiagnosed, particularly when neurologic involvement is mild or absent [42].

#### Cardiovascular manifestations

In both hereditary and nonhereditary TTR amyloidosis, as in amyloid light-chain (AL) amyloidosis, amyloid can infiltrate any or all of the cardiovascular structures, including the conduction system, the atrial and ventricular myocardium, valvular tissue, and the coronary and large arteries [38,39,43]. The conduction system is commonly affected, leading to bundle branch block and, occasionally, atrioventricular (AV) and sinoatrial block. Myocardial infiltration progressively increases the thickness of the left and right ventricular walls and the interventricular septum. Cardiac amyloidosis is generally considered to be a cardiomyopathy with a hypertrophic phenotype and restrictive pathophysiology, although a true restrictive filling pattern is seen only in advanced stages [44]. Left ventricular (LV) ejection fraction is normal or only mildly reduced. However, abnormalities in long-axis function of both ventricles on tissue Doppler imaging are frequent and precede the impairment of circumferential ventricular function [45]. The involvement of the cardiac valves leads to the formation of nodules or diffuse thickening of the leaflets, accompanied by variable degrees (generally mild) of valvular regurgitation. The clinical spectrum of cardiovascular involvement is wide, ranging from asymptomatic AV and bundle branch block to severe, rapidly progressive heart failure due to restrictive pathophysiology. These variations are related to the specific *TTR* mutation, geographic area, and endemic/non-endemic demographics of individual cases.

#### When should a cardiologist suspect TTR amyloidosis?

Cardiologists may encounter TTR amyloidosis in patients who are referred with neurologic impairment or previously diagnosed TTR-FAP, and in patients who present with cardiologic problems without any apparent signs of neurologic disease. If there is a strong suspicion or an existing diagnosis of TTR-FAP, the cardiologist should look for signs of cardiac amyloid involvement (Appendix B). In such situations, electrocardiography (ECG), echocardiography, scintigraphy with bone tracers, biomarkers (brain natriuretic peptide [BNP] and troponin I or T) and cardiac magnetic resonance imaging usually provide all the necessary information to diagnose infiltrative cardiomyopathy. The study of longitudinal LV function with tissue Doppler echocardiography and ^99m^Tc-3,3-diphosphono-1,2-propanodicarboxylic acid (^99m^Tc-DPD) myocardial scintigraphy are particularly revealing for very early signs of myocardial involvement [45-47]. Although as a general rule histological evidence of amyloid deposits is essential for a final diagnosis of amyloidosis, in a patient with molecularly proven diagnosis of TTR amyloidosis, identification of unexplained ventricular hypertrophy and other typical instrumental findings (see below) provides convincing evidence of amyloidotic cardiomyopathy.

When the phenotypic expression of TTR amyloidosis is exclusively or predominantly cardiac, the situation is far more challenging. Affected patients may present for a wide variety of reasons, including symptoms of heart failure, arrhythmias, syncope, orthostatic hypotension, and ECG/echocardiographic abnormalities in the absence of symptoms. The problem of differential diagnosis from cardiomyopathies of other causes is common, and a frequent pitfall is the misdiagnosis of cardiac amyloidosis as sarcomeric hypertrophic cardiomyopathy. A particularly challenging situation regards elderly African-Americans with hypertension, increased LV wall thickness, and minimal or no neuropathy, since a diagnosis of TTR amyloidosis in such patients may be overlooked. A logical first step is to look for the infiltrative phenotype with echocardiography and for any major discrepancy between the assessment of LV mass with echocardiography and ECG (for example, when increased mass via echocardiography is not accompanied by ECG signs of LV hypertrophy) [38,42]. Another useful clue can be the recognition of right-sided heart failure, unusual in hypertensive heart disease [42]. ^99m^Tc-DPD scintigraphy can assist in the differential diagnosis between TTR-related cardiomyopathy and sarcomeric hyptertrophic cardiomyopathy, since only myocardium infiltrated by TTR uptakes the tracer [48].

Apart from evident neurologic manifestations, some clinical signs can raise the suspicion of hereditary TTR amyloidosis in a patient with apparently isolated hypertrophic cardiomyopathy. These signs include a history of carpal tunnel syndrome, sensorimotor peripheral neuropathy, unexplained intense myalgias and burning sensations, and autonomic dysfunction. Careful evaluation for these signs is mandatory, as they can sometimes be very mild and may not be reported by the patient.

### Leptomeningeal TTR amyloidosis

Attention has recently focused on the relatively rare leptomeningeal form of TTR amyloidosis, which is induced by several point mutations in the *TTR* gene and also found in the advanced stages of Val30Met TTR-FAP [37]. In most cases, it is believed that the source of variant *TTR* in leptomeningeal amyloidosis is not the liver but the choroid plexus [49]. Cerebral amyloid angiopathy and ocular amyloidosis are common clinical features of this type of TTR amyloidosis. Cerebral amyloid angiopathy is characterized by amyloid deposition in the media and adventitia of medium-sized and small arteries, arterioles, and, occasionally, veins of the cortex and leptomeninges. Typical clinical central nervous system manifestations include cerebral infarction and hemorrhage, hydrocephalus, ataxia, spastic paralysis, convulsion, and dementia. These symptoms are often found in several types of TTR amyloidosis and they lead to the classification of (oculo)leptomeningeal amyloidosis, in which amyloid deposition is also found in vitreous bodies and other tissues of the eye. Although amyloid deposits in the meningocerebrovascular system are thought to be the cause of those central nervous system symptoms, the precise mechanism of amyloid formation remains to be elucidated.

### Other organ involvement

Gastrointestinal symptoms, as a manifestation of autonomic neuropathy, are recognized from an early stage of TTR-FAP and include nausea, early satiety, recurrent vomiting, watery diarrhea, severe constipation, and/or alternating diarrhea and constipation. As a result, weight loss may be observed in the course of the disease. Renal complications, while infrequent, include albuminuria and/or mild azotemias. Dry eye is an early autonomic symptom of TTR-FAP, and a decrease in visual acuity induced by vitreous opacity or glaucoma is recognized as TTR-FAP progresses.

## Tests and assessments

### Overview

Neurologic examination should indicate the presence or absence of a length-dependent sensorimotor axonal neuropathy affecting temperature and pain detection in the feet. Ultimately a patient should undergo a complete neurologic examination, which may include electromyographic testing with sympathetic skin response (SSR), quantitative sensory testing, heart rate deep breathing, and other autonomic tests, determined by presenting physical signs. Cardiac evaluation should include ECG, echocardiography, BNP/troponin measurement and, in select cases, cardiac magnetic resonance, scintigraphy with bone tracers, and Holter monitoring. Following this workup the patient should undergo a DNA analysis and tissue biopsy (Table 1), although the uneven distribution of amyloid fibrils may yield false-negative results. While experienced neurologists in endemic areas may be able to diagnose TTR-FAP by the presence of reported family history of the disease as well as relevant symptoms alone, a biopsy showing amyloid deposits is mandatory in patients without a family history (late-onset and apparently sporadic forms) and in populations with a low disease prevalence. Genetic testing is needed to document the pathogenic mutation. If it is normal, TTR-FAP is excluded. Symptoms and stage of disease progression can be identified by neurologic tests and the modified body mass index (mBMI; BMI multiplied by serum albumin level [g/L] to compensate for edema formation) [50], a measure of nutritional status and wasting.

### Diagnosis

#### Tissue biopsy

To confirm amyloidosis, the demonstration of amyloid deposits via tissue biopsy is essential. Deposition of amyloid in the tissue can be demonstrated by Congo red staining of biopsy specimens [51]. With Congo red staining, amyloid deposits show a characteristic green birefringence under polarized light. Tissues suitable for biopsy include subcutaneous fatty tissue of the abdominal wall, kidney, skin, gastric, or rectal mucosa; sural nerve tissue; retinaculum and peritendinous fat obtained at carpal tunnel surgery; and tissue from the salivary gland. The sensitivity of endoscopic biopsy of the gastrointestinal mucosa is approximately 85%, whereas biopsy of the sural nerve is less sensitive because amyloid deposition is often sporadic and random [52]. TTR immunolabeling of the amyloid deposits can identify the disease as TTR amyloidosis but cannot distinguish wild-type from hereditary forms. In patients with typical signs and symptoms of TTR amyloidosis, negative biopsy results should not be interpreted as excluding the disease.

#### Serum variant TTR protein

TTR protein normally circulates in serum or plasma as a soluble protein with a tetrameric structure. The normal plasma TTR concentration is 20 to 40 mg dL^–1^ (0.20 to 0.40 mg mL^–1^). After immunoprecipitation with anti-TTR antibody, serum variant TTR protein can be detected by mass spectrometry [53]. Approximately 90% of TTR variants identified exhibit the mass shift predicted by the one amino acid substitution of the variant TTR [54].

#### Genetic confirmation

Although mass spectrometry can demonstrate a mass difference between wild-type and TTR protein variants in serum, it does not specify the site and kind of amino acid substitution in a number of disease-related *TTR* gene mutations; thus, DNA sequencing is usually required. Current techniques for performing sequence analysis of *TTR*, the only gene known to be associated with TTR amyloidosis, detect >99% of disease-causing mutations.

### Exclusionary diagnoses

#### AL amyloidosis

The diagnosis of AL amyloidosis is often considered due to the high incidence of monoclonal gammopathies in the elderly. Differentiation of AL and TTR amyloid polyneuropathy requires analysis of the subunit protein comprising amyloid tissue deposits. Amyloid immunohistochemical staining has been the standard approach; however, the antibody staining can be misleading because of suboptimal antibody reagents or excessive background staining. In contrast, immunoelectron microscopy may provide a clear-cut characterization of amyloid fibrils, but this technique is not widely available. Mass spectrometry-based proteomic analysis or immuno-gold electron microscopy can be useful in this setting, but *TTR* genopositivity should be established by DNA analysis in all cases of suspected FAP.

#### Mimicking neuropathies

Some adult patients with diabetes mellitus may develop a polyneuropathy similar to TTR-FAP with early and predominant small-fiber involvement and autonomic dysfunction. Chronic alcoholism can also induce a peripheral neuropathy that is indistinguishable from TTR-FAP disease.

#### Chronic inflammatory demyelinating polyneuropathy (CIDP)

When a family history is not known, a diagnosis of CIDP is often considered first. In CIDP, large myelinated fiber dysfunction predominates, with slow conduction velocity, high cerebrospinal fluid (CSF) protein content, and virtually no symptoms of autonomic dysfunction. However, axonal lesions predominate in some cases. In addition, nerve conduction velocity is often mildly decreased in patients with TTR-FAP, and CSF protein content can be elevated, which may increase the confusion with CIDP [40]. In such cases, a nerve biopsy may differentiate amyloid from CIDP by revealing congophilic deposits.

#### Other exclusions

Other diagnoses to exclude are familial amyloid polyneuropathy (gelsolin type, apolipoprotein A1 type), other types of inherited sensory polyneuropathy, hereditary sensory and autonomic neuropathies, Fabry’s disease, leprous neuropathy, anxiety, irritable bowel syndrome, cardiac disorders (e.g. arrhythmias, cardiac failure), ocular disorders (e.g. macular degeneration), alcoholism, and vitamin B12 deficiency.

### Tests for neuropathic symptoms

Neuropathic symptoms can be assessed by the polyneuropathy disability (PND) score; neuropathy symptom score; neurologic disability score (NDS); neuropathy impairment score (total NIS), and neuropathy impairment score–lower limb (NIS-LL), scales that quantify neurologic function in patients with diabetic polyneuropathy but are also valuable for patients with TTR-FAP; autonomic reflex screen; composite autonomic severity score; mBMI; electromyography with SSR; and other original scores or local variants.

### Tools for evaluating TTR-FAP progression

The progression of TTR-FAP can be evaluated by nerve conduction velocity, sensory action potentials, motor action potentials, SSR amplitude, NDS, NIS-LL, and quantitative sensory testing for characterizing small-fiber (coolness and heat detection) and large-fiber (vibratory detection) peripheral sensory thresholds. Autonomic function is assessed by heart rate deep breathing measures. Other means of assessing the progression of TTR-FAP include echocardiography, Holter monitoring, ophthalmologic testing, mBMI measurement, and electrophysiologic evaluation. Measurements should be made every 6 months.

Laboratory examinations for evaluating TTR-FAP progression include measuring cardiac (plasma BNP, NT-proBNP, and high-sensitivity serum troponin concentration) and renal (creatinine clearance and albuminuria) parameters.

### Scoring systems

Several scoring systems for evaluating TTR-FAP have been proposed from various clinical centers (Appendix C). These include systems based on the stages of peripheral and autonomic neuropathies proposed by Coutinho et al. [55], disease staging based on PND score, the Portuguese classification to evaluate the severity of TTR-FAP [56], sensory impairment scoring, autonomic dysfunction scoring, and scoring of motor function for muscle weakness.

## Disease-modifying treatments for ttr-fap

Current treatment options for patients with TTR amyloidosis are limited. For patients with TTR-FAP who have mild or moderate disease and a diagnosis confirmed by genetic testing and biopsy, liver transplant is the current standard of care. However, symptomatic treatment to provide immediate relief is a priority (Table 2). Since variant TTR is produced largely in the liver, transplanting a new liver should almost completely eliminate production of the variant protein and halt disease progression outside the brain and eyes. However, liver transplant does not effectively prevent cardiomyopathy in most cases and is not recommended for patients with late-stage TTR-FAP or with leptomeningeal-type amyloidosis. For these patients, symptomatic relief is the only treatment strategy at present. The first pharmacologic agent for patients with TTR-FAP was approved in Europe in late 2011, and several other agents are in various stages of clinical development. These pharmacotherapies are described in more detail below.

**Table 2:** Treatment for clinical symptoms of transthyretin familial polyneuropathy (TTR-FAP)

**Symptom**	**Treatment**
Arrhythmias	Pacemaker implantation, pharmacotherapy
Cardiac failure	Diuretics, angiotensin converting enzyme inhibitors
Orthostatic hypotension	Droxidopa, midodrine, amezinium metisulfate, fludrocortisone, plastic stocking, abdominal belt, elevating head
Gastrointestinal disorders (not severe)	Polycarbophil calcium, metoclopramide
Severe diarrhea	Loperamide
Neuropathic pain	Pregabalin, gabapentin, amitriptyline, duloxetine
Carpal tunnel syndrome	Surgery
Dry mouth	Potassium dihydrogen phosphate, cevimeline
Hypoglycemia	Glucose loading
Renal failure	Hemodialysis
Urinary incontinence	Distigmine
Anemia	Erythropoietin, iron
Hypothyroidism	Levothyroxine
Ocular amyloidosis	Vitrectomy, trabeculectomy

### Liver transplant

Orthotopic liver transplant is the only disease-modifying treatment available to patients with TTR-FAP. This removes roughly 95% of the production of variant TTR and can slow or halt the progression of the disease. In most countries liver transplant is performed using organs from deceased donors, but in Japan liver tissue from live donors is used more frequently. Liver transplants for the treatment of TTR-FAP are reported by participating centers to the Familial Amyloidotic Polyneuropathy World Transplant Registry (FAPWTR, Appendix A).

Nerve function rarely improves after successful liver transplantation; however, a lessening of autonomic disturbances may occur. It is generally agreed that the best outcomes achieved with liver transplant occur when the procedure is performed in young patients whose disease has not become advanced. Notably, some patients do not perceive an improvement in their quality of life (QOL) although their disease progression has been halted and survival improved.

To be eligible for liver transplant, a patient should have genetic proof of TTR-FAP, biopsy proof of amyloid deposits, and symptoms of early-stage TTR-FAP. Liver transplantation in later stages (e.g. PND stage II or III; see Appendix C) may be complicated by progressive amyloid cardiomyopathy or neuropathy, prompting many amyloid experts to recommend liver transplant only in patients with early disease.

#### Outcomes of liver transplant for patients with TTR-FAP

Many patients have benefitted from liver transplant and are able to live relatively normal lives, although the organ impairment that occurred before the transplant does not usually reverse. Long-term observations of transplanted patients have clearly shown the histopathologic regression of amyloid deposits [54] with an overall patient survival rate at 5 years of >77% [57]. Current data from the FAPWTR indicate a 10-year survival rate of 74% for Val30Met versus 44% for non-Val30Met patients, emphasizing mutation-specific utility of liver transplantation in TTR-FAP [57]. In addition, some patients continue to demonstrate disease progression following liver transplant. Mounting evidence indicates that TTR-FAP patients with significant amyloid infiltration of the heart or nerves at the time of liver transplantation may experience progression of disease postoperatively due to wild-type TTR deposition on existing amyloid of variant TTR [58]. In addition to the risks of surgery, transplanted patients also need to remain on immunosuppressants for the rest of their lives.

Prognosis after liver transplant is influenced by many factors, including the properties of particular TTR variants, nutritional status, age, and severity of neuropathy and cardiac amyloid involvement. Studies of survival as well as physical and mental improvement after transplant are still needed, especially for patients with a non-Val30Met mutation.

#### Domino liver transplant (DLT)

The cornerstone of transplantation is organ availability. Despite advances in this field, a significant proportion of patients with liver failure may die without a chance at liver replacement. Because liver involvement in TTR-FAP is minimal, the liver of an affected individual can be grafted into selected patients (without TTR-FAP) who meet the criteria for transplant but for whom the current allocation schemes for cadaveric organs may not allow transplant within a reasonable time. This procedure has been termed DLT. Since 1995, more than 950 DLTs have been performed worldwide [59]. The assumption was that amyloidosis would not appear for several decades after liver transplant because patients rarely become symptomatic before the age of 20 years and most often not until their forties or fifties [60]. However, *de novo* systemic amyloidosis after DLT was found to appear in a small number of patients earlier than initially expected, and a number of DLT recipients have since been retransplanted due to the transmission of TTR-FAP through a domino graft 8 to 10 years after the initial transplant [61]. Also, subclinical cutaneous TTR deposits were reported in five other recipients of DLT grafts [61,62]. Despite this, the donor shortage still justifies DLT in selected patients. Post-transplant surveillance for amyloid disease requires tissue biopsy and electromyography in combination with observation for clinical manifestations. Recipients of DLTs need to be monitored for signs of *de novo* amyloid disease after transplant. DLTs are also reported to the FAPWTR, within the Domino Liver Transplant Registry (Appendix A).

#### Combined heart and liver transplant

Cardiac risks and complications constitute major adverse events in patients undergoing orthotopic liver transplant for TTR-FAP. Cardiovascular complications account for about 39% of deaths following liver transplant, almost half of which occur within the first 3 months [57,63]. Moreover, cardiac disease may progress even after successful liver transplant, especially in patients with mutations other than Val30Met, due to the deposition of wild-type TTR fibrils on preexisting amyloid matrix. In highly selected patients these considerations may provide a rationale for the extremely challenging procedure of combined heart and liver transplant [60]. The main indication for this procedure is severe heart failure due to amyloidotic cardiomyopathy in a patient without advanced neurologic involvement. It has also been proposed as a therapeutic option for patients affected by mutations other than Val30Met who are candidates for liver transplant and who have an echocardiographic diagnosis of cardiomyopathy even in the absence of major cardiovascular symptoms [41,60].

### Pharmacotherapy

Elucidation of the mechanisms contributing to TTR misfolding and fibril formation identified TTR-tetramer stabilization as a rate-limiting event, leading to the development of several new pharmacologic therapies for patients with TTR-FAP. TTR stabilizing agents can be prescribed at an early stage of disease in anticipation of liver transplantation or, potentially, delaying the need for liver transplant. Ongoing and recently completed clinical trials investigating several pharmacotherapies are described in Appendix D.

#### Tafamidis

Tafamidis (Vyndaqel®; Pfizer Inc, New York, NY, USA) is a disease-modifying agent that kinetically stabilizes TTR [64]. It limits dissociation of the native TTR tetramer into monomers, a critical step in fibril generation, inhibiting TTR amyloid fibril formation. The pivotal phase 2/3 trial showed that tafamidis was well-tolerated, and although the coprimary efficacy endpoints (response to treatment at Month 18 as measured by NIS-LL and Norfolk Quality of Life–Diabetic Neuropathy total score) were not met, the totality of the results suggested that tafamidis could slow progression in patients with stage I disease. Although the study only included patients aged 18 to 75 years, there is no age limit for initiating patients on tafamidis. Data show that tafamidis is well tolerated in patients with stage I disease, although gastrointestinal side effects and urinary tract and vaginal infections may occur. Tafamidis has orphan drug designation for TTR-FAP in the United States and Europe. In 2011, the European Medicines Agency approved tafamidis for use in Europe for patients with early (stage I) Val30Met and non-Val30Met TTR-FAP. The FDA has requested additional clinical data through a second clinical trial before considering approval in the United States. There are no data supporting the treatment of patients with stage II or III TTR-FAP or those who have familial amyloid cardiomyopathy. Such patients should only receive tafamidis within a clinical trial protocol.

#### Diflunisal

Diflunisal (Dolobid®; Merck and Co, Inc, Whitehouse Station, NJ, USA) is a commercially available nonsteroidal anti-inflammatory agent that stabilizes TTR tetramers *in vitro*, preventing disaggregation, monomer release, and amyloid fibril formation by misfolded TTR monomers. A phase 1 study demonstrated that low doses of diflunisal stabilized TTR tetramers with no toxicities; and a phase 2/3 clinical trial involving 130 TTR-FAP patients will conclude in December 2012 [65]. The risks of gastrointestinal bleeding, altered renal function, or fluid retention may be low in a study population, but patient selection and surveillance for adverse events are important components of treatment.

#### ALN-TTR01 and ALN-TTR02

ALN-TTR01 and ALN-TTR02 (Alnylam Pharmaceuticals, Inc, Cambridge, MA, USA) are systemically delivered RNA interference therapeutics designed to suppress the expression of TTR protein and prevent amyloid formation. ALN-TTR01 employs a first-generation lipid nanoparticle formulation. Studies of ALN-TTR01 in TTR-FAP transgenic mice have shown both prevention and regression of pathogenic TTR deposits in peripheral tissues, while a single dose results in rapid, dose-dependent, and sustained reduction in serum TTR levels in patients with Val30Met TTR-FAP. Recently, Alnylam announced results of a phase 1 single-dose escalation trial of ALN-TTR02, a second-generation lipid nanoparticle with >10-fold milligram potency in suppressing serum TTR expression versus ALN-TTR01. Phase 2 studies are underway.

#### ISIS-TTRRx

ISIS-TTRRx (Isis Pharmaceuticals, Inc, Carlsbad, CA, USA) is an antisense therapy that suppresses *TTR* mRNA expression. A phase 1 study demonstrated a dose-dependent reduction of TTR in healthy patients with no reported significant adverse events [66]. A phase 2/3 study examining the effect of the anti-sense oligonucleotide on disease progression and QOL in patients with TTR-FAP is scheduled to begin in 2012.

#### Doxycycline/tauroursodeoxycholic acid (Doxy-TUDCA)

The combined use of doxycycline, an antibiotic that disrupts TTR amyloid fibril formation, and TUDCA, a drug used in patients with liver disorders that can reduce nonfibrillar TTR deposition, has demonstrated a synergistic effect on lowering TTR deposits in mouse models of TTR-FAP [67]. A phase 2 open-label trial evaluating the pharmacokinetics, efficacy, safety, and tolerability of Doxy-TUDCA is ongoing [68].

## Symptom management of ttr-fap

### Staging overview (based on Coutinho [55])

Patients with stage 0 disease are asymptomatic but have both a variant form of the *TTR* gene and evidence of amyloid deposits. Patients with stage I (mild) disease are ambulatory, patients with stage II (moderate) disease are ambulatory but require assistance, and patients with stage III (severe) disease are bedridden or wheelchair-bound.

#### Stage 0 disease

As disease onset is often late and diagnosis is difficult, for many patients sometimes a definitive diagnosis is made only in their sixties or seventies, although some patients are diagnosed in their thirties or earlier. In some countries, such as Portugal, patients tend to be young at diagnosis, but in all cases these patients are likely to have offspring or even grandchildren who may want to be tested for *TTR* gene mutation. Offspring who are positive for the mutated gene and test positive for amyloid deposits are considered to have stage 0 disease. Patients with stage 0 disease could be considered candidates for pharmacotherapy, but because there are no data to support the efficacy and safety of treatment for such patients, they should be treated only within the confines of a clinical trial.

#### Stage I disease

It is important to emphasize that, as with many diseases, early detection is critical and can lead to the best outcomes. From the outset, patients should be treated symptomatically and stabilized (Table 2). Since the only widely available, approved treatment for TTR-FAP is liver transplant, all patients with stage I disease, irrespective of mutation status or age, should be placed on a liver transplant list. Early liver transplant is important, and younger patients with mild symptoms are the best candidates.

Concurrently, patients should be treated with any approved drugs as they become available and as the patient’s disease state meets drug indications, independent of liver transplant plans. Small-molecule inhibitors, such as tafamidis and diflunisal, may prolong the time from disease onset to progression. Future studies will determine whether one or both of these agents will have durable inhibitory effects on TTR amyloidotic neuropathy and cardiomyopathy.

To be eligible for treatment, symptomatic patients should have a confirmed diagnosis of TTR-FAP. Genetic documentation as well as a positive amyloid biopsy would be a sufficient confirmation of diagnosis. Patients living in highly endemic areas of the world with a *TTR* mutation and typical symptoms, depending on the conventions of the medical community, may not require a positive biopsy for treatment consideration (sites of amyloid deposits can vary, so not all will be positive on biopsy). However, if there is no biopsy evidence of amyloid deposition, confounding diagnoses, such as pain due to diabetic peripheral neuropathy, need to be excluded.

Patients who have been placed on pharmacotherapy with objective evidence of disease progression should pursue other treatment options such as liver transplant. For patients exhibiting disease stabilization on pharmacotherapy, there is currently no consensus among experts regarding the utility of liver transplant, as long-term pharmacologic data are not available to address treatment durability.

Follow-up assessments should be performed on an average of every 6 months by an experienced physician to identify disease progression. This is particularly important for patients who have early and/or substantial cardiac involvement. The actual battery of assessments to follow patients should, at a minimum, mirror the Transthyretin Amyloidosis Outcomes Survey (THAOS) evaluation (full sensory and motor evaluation and echocardiography) and include the NIS-LL (which can be measured by nonexpert physicians), mBMI, and electroneuromyography.

#### Stage II disease

Data supporting the use of disease-modifying pharmacotherapy in patients with stage II disease are not available at this time. Market approval for tafamidis in Europe is limited to patients with stage I disease. Patients with early stage II disease may also be considered for liver transplant. Whether TTR-stabilizing pharmacotherapy will prove effective in slowing or stopping disease progression in patients with stage II disease requires further clinical study. These patients, as well as patients with stage II disease who demonstrate cardiac involvement, should receive disease-modifying pharmacotherapy only within the confines of a clinical trial.

#### Stage III disease

While it is theoretically possible that the pharmacologic treatment of patients who have stage III disease might prolong or improve their QOL, no data are available to support this. Such patients should receive disease-modifying pharmacotherapy only within the confines of a clinical trial.

### Liver transplant recipients

The pharmacologic treatment of liver transplant recipients may be appropriate, but currently there are no data available to support the use of disease-modifying therapies in these patients. A phase 2 study of combined doxycycline/TUDCA for the treatment of patients with TTR amyloidosis includes liver transplant recipients (Appendix D).

### DLT recipients

Although a rationale exists for treating DLT recipients with pharmacotherapy, no data are available to support this practice. Drug treatments should not be considered without evidence of amyloid deposition (e.g. biopsy proof). At present, DLT recipients should be treated only in a clinical trial.

## Patient support

All efforts should be made to identify TTR-FAP early. Once patients have developed stage I disease they should be treated symptomatically regardless of disease presentation. The goal is to alleviate symptoms and subsequently establish a long-term strategy. Tafamidis, diflunisal, or any other treatments shown to be efficacious should be started at once. Until more data are available, patients with later-stage disease should be treated only within clinical trials. The questions of early prophylaxis and posttransplant administration have yet to be formally addressed but offer rich possibilities for further research.

For patients requiring liver transplant, local regulations covering eligibility for transplant vary widely, with age limits in some countries among other restrictions. Physicians should discuss all possible options with their patients. It is not clear whether guidelines for transplant will change once new medications are approved.

TTR-FAP is extremely difficult to manage; thus, patients and their families need all the social and moral support possible. It is important to stress that the physician involved should be a specialist in peripheral nerve disease and be experienced in alleviating the symptoms associated with neuropathies. Some countries will have specific centers of excellence, but this is not the case everywhere.

### Genetically related family members

Genetic testing should be performed on relatives of patients with TTR-FAP when they are capable of comprehending the medical, social, and psychological ramifications of a positive result. Relatives should be strongly encouraged to undergo genetic testing and, if positive, tissue biopsies. Treatment options can then be considered. However, for some individuals, including those with a family history of late-onset disease, early genetic testing may produce severe anxiety to which the physician needs to be sensitive. In all cases, genetic counseling and, if necessary, psychological support should be offered to affected family members. In some countries long-term insurance eligibility is affected by genetic disease and should be considered before DNA analysis.

### Disease registries

Physicians are encouraged to enter their patients into the appropriate registry or registries. These registries act as valuable resources for disease awareness (for both the public and the provider), inform clinical practice habits, and identify gaps in knowledge. Information on enrollment can be found on the websites listed in Appendix A.

#### THAOS (Transthyretin Amyloidosis Outcomes Survey)

THAOS, an international, longitudinal, observational survey, developed by FoldRx Pharmaceuticals, Inc, (and now supported by Pfizer Inc) in collaboration with clinical disease experts, is designed to characterize the variability, progression, and natural history of TTR amyloidosis, as well as regional differences in disease expression and the genotypic/phenotypic relationship in TTR amyloidosis. This ongoing survey seeks to gather information on patients diagnosed with TTR amyloidosis to better understand this rare disease. As of March 2012, a total of 1224 individuals (657 male and 567 female) had enrolled at 46 sites in 19 countries. Of these, 1111 individuals had *TTR* mutations, of whom 834 had the Val30Met mutation and 781 were symptomatic.

#### FAPWTR

The FAPWTR monitors the history of patients with TTR-FAP who had undergone liver transplant. As of December 31, 2011, a total of 1985 liver transplant recipients were recorded in the FAPWTR, including 37 combined heart-liver transplants. Currently 73 centers perform liver transplant in 19 countries [59]. The objectives of the FAPWTR are to promote the collaboration and exchange of experience, monitor international transplant activity, help optimize patient selection and ensure adequate follow-up after transplant, and function as an investigative tool for reporting centers.

#### Domino liver transplant registry

The Domino Liver Transplant Registry documents patients who have received a liver from a patient affected with amyloidosis. Originally it was believed that symptoms would take decades to develop, but recently some patients have become diseased much sooner.

## Conclusion

TTR amyloidosis is a progressive and fatal disease that is increasingly diagnosed worldwide. Previously, liver transplant was the only available treatment for patients with TTR-FAP; the approval of tafamidis in 2011 for use in Europe in patients with stage I TTR-FAP has broadened therapeutic options.

The most reliable diagnostic approach involves genetic testing followed by a tissue biopsy to confirm active amyloid formation. Common symptoms also exist for which there are assessment and scoring tools that are important for documenting disease progression. Most importantly, patients should be treated symptomatically regardless of presentation and, if possible, stabilized, since the immediate goal is to alleviate symptoms. Physicians should also probe for certain autonomic symptoms, such as irritable bowel syndrome and erectile dysfunction, which may not always be revealed on a standard examination. Such symptoms can then be addressed. All patients with early-stage disease should be evaluated for liver transplant and concurrently considered for drug treatment. Patients with later-stage disease or cardiomyopathy have not yet been shown to benefit from pharmacotherapy. The decision to remain on the transplant list, if improvement is seen with drug treatment, depends on local rules and on each individual case. To help make this decision, physicians should monitor their patients every 6 months with full sensory and motor evaluation, echocardiography, NDS, and mBMI.

Many questions remain, including how to treat asymptomatic patients with the TTR-FAP Val30Met mutation and patients with stage 0 disease who have documented amyloid, as there are no data to support prophylactic treatment. In addition, there are no recommendations regarding pharmacotherapy for liver transplant or DLT recipients. Current and upcoming clinical trials will provide a basis for the proper administration of new drug treatments and improved guidance for the use of currently available agents.

## Appendix A

### Recommended reading

Benson MD, Kincaid JC: The molecular biology and clinical features of amyloid neuropathy. *Muscle Nerve* 2007, 36:411–423.

Coelho T, Ericzon B-G, Falk R, et al.: A physician’s guide to transthyretin amyloidosis. Available at http://www.amyloidosis.org/pdf/TTR%2008.pdf. Last updated August 2008.

Falk RH: Diagnosis and management of the cardiac amyloidoses. *Circulation* 2005, 112: 2047–2060.

Herlenius G, Wilczek HE, Larsson M, Ericzon BG: Ten years of international experience with liver transplantation for familial amyloidotic polyneuropathy: results from the Familial Amyloidotic Polyneuropathy World Transplant Registry. *Transplantation* 2004, 77:64–71.

Jacobson DR, Pastore RD, Yaghoubian R, et al.: Variant-sequence transthyretin (isoleucine 122) in late-onset cardiac amyloidosis in black Americans. *N Engl J Med* 1997, 336:466–473.

Planté-Bordeneuve V, Said G: Familial amyloid polyneuropathy. *Lancet Neurol* 2011; 10: 1086–97.

Sekijima Y, Yoshida K, Tokuda T, et al.: Familial Transthyretin Amyloidosis. 2001 Nov 5 [Updated 2012 Jan 26]. In: Pagon RA, Bird TD, Dolan CR, et al., editors. GeneReviews™ [Internet]. Seattle (WA): University of Washington, Seattle; 1993-. Available from: http://www.ncbi.nlm.nih.gov/books/NBK1194/

### Helpful websites

Amyloidosis Foundation (http://www.amyloidosis.org)

ClinicalTrials.gov (http://www.clinicaltrials.gov)

Familial Amyloidotic Polyneuropathy World Transplant Registry and Domino Liver Transplant Registry (http://www.fapwtr.org)

Transthyretin Amyloidosis Outcomes Survey (http://www.thaos.net)

## Appendix B

### Recommended studies for TTR amyloidosis patients with cardiomyopathy

#### ECG and echocardiographic examinations

The preliminary workup for a patient with suspected cardiac amyloidosis includes 12-lead ECG and two-dimensional echocardiography. Echocardiography provides the mainstay for the noninvasive diagnosis of amyloidotic cardiomyopathy. A key point is to consider not only LV wall thickness but other findings that could suggest cardiac infiltration. Based on a study of patients with a definite diagnosis of AL amyloidosis, Gertz et al. [69] suggested a composite echocardiographic approach based on the presence of end-diastolic thickness of the interventricular septum >1.2 cm (in the absence of any other cause of ventricular hypertrophy) plus two or all of the following: homogeneous AV valve thickening, atrial septum thickening, and sparkling/granular appearance of the ventricular septum. This has also proved to be valid for assessing other etiologic forms of amyloidotic cardiomyopathy [70]. Pericardial effusion is another frequent finding in cardiac amyloidosis.

The combination of pericardial effusion and increased ventricular wall thickness should prompt a strong suspicion of amyloidotic cardiomyopathy. In such cases, the ventricles are usually not dilated and LV ejection fraction tends to be normal or only slightly reduced, but the wall-thickening velocity is frequently depressed [71]. Similarly, the long-axis function of the left ventricle is often reduced even in the early stages of the disease when radial fractional shortening is still conserved [72,73].

Disease profile and instrumental features (as well as clinical outcome) are related to the specific etiologic form of cardiac amyloidosis, and profound differences exist among the three most frequent pathogenic types of amyloidotic cardiomyopathy (AL, wild-type, and mutant TTR amyloidosis). In a large series of patients with amyloidotic cardiomyopathy, the mean value of wall thickness was, on average, lower in patients with AL than in patients with hereditary TTR or senile systemic amyloidosis, although the overlapping distributions seem to preclude the use of this measure for differential diagnosis [70]. Conversely, echocardiographic features suggestive of amyloidotic involvement commonly appear only in the later stages of the disease [38,72]. Thus, echocardiographic images cannot be used in isolation for diagnostic confirmation and must be interpreted in the context of the clinical picture and other examinations. Perhaps the single most useful sign is the presence of low or normal QRS voltage despite increased LV wall thickness. Unfortunately, this highly specific sign is not very sensitive, and the prevalence of low QRS voltage (QRS voltage amplitude ≤0.5 mV in all limb leads or ≤1 mV in all precordial leads) usually is significantly lower in patients with TTR amyloidosis than in patients with AL [70]. This observation underscores the importance of pursuing any clinical suspicion of TTR amyloidotic cardiomyopathy, even in the absence of reduced QRS voltage.

Other common ECG abnormalities include repolarization alterations; anterior, inferior, or lateral pseudoinfarct patterns; left anterior hemiblock; bundle branch block; ischemic-type or nonspecific T-wave abnormalities [74,75], and rhythm disturbances (e.g. atrial fibrillation). A low-voltage ECG with increased septal and posterior LV wall thickness on echocardiography is specific for cardiac amyloidosis on noninvasive testing [74,75].

#### MRI

MRI with delayed gadolinium enhancement has proved very useful for the detection of amyloid protein in the myocardial interstitium. Since gadolinium tends to accumulate within the interstitium infiltrated by amyloid, in the presence of amyloidotic cardiomyopathy MRI provides two highly specific findings: alterations in gadolinium kinetics, with a faster-than-normal washout of gadolinium from the blood and myocardium, and a late enhancement effect, often with a predominant diffuse, global, and subendocardial distribution [76], although in some cases accumulation of amyloid in the myocardial interstitium may appear more focal with variable transmural extension [77].

Current MRI studies collectively describe patients with different types of systemic amyloidosis, including TTR amyloidosis, who exhibit previous diagnostic signs common to all types of amyloidosis. Therefore, it is unknown whether MRI has the capability to distinguish between amyloid types.

#### Scintigraphy

Myocardial amyloid involvement can be visualized by several scintigraphic tracers, each of which binds to distinct parts of amyloid fibrils [78]. ^123^I-labeled serum amyloid P (SAP) protein binds in a calcium-dependent way to all amyloid deposits, irrespective of the protein of origin. SAP scintigraphy (which is available only in a few highly specialized centers) provides a reliable semiquantitative evaluation of the extent and distribution of amyloid deposits in the soft tissues and visceral organs, but not specifically in the heart [79-81].

^99m^Tc-aprotinin, a protease inhibitor, is able to detect extra-abdominal amyloid deposits, such as cardiac deposits, thanks to the physiologic uptake of the tracer in the liver, spleen, and kidneys [82,83]. However, available experience is limited and quantitative assessment is not feasible [79,82-84]. One tracer that has been used for detecting cardiac amyloid deposition is ^123^I-metaiodobenzylguanidine (MIBG). Although ^123^I-MIBG does not bind directly to amyloid deposits, ^123^I-MIBG scintigraphy has proven useful in the functional evaluation of amyloid cardiomyopathy because it indirectly reveals the impairment of cardiac sympathetic nerve endings due to amyloid deposition [79,85,86]. There is increasing evidence that ^99m^Tc-DPD scintigraphy can image amyloid deposition in the myocardium with a greater sensitivity among patients with TTR-related cardiac amyloidosis (both mutant and wild-type) than immunoglobulin AL-related amyloidosis, and that, compared with a small series of normal controls, it is a highly specific imaging tool that can facilitate the differential diagnosis between TTR and AL cardiac amyloidosis in routine practice [47,87].

## Appendix C

### Scoring methods

#### Clinical staging of TTR-FAP (based on Coutinho et al.)

• Stage 0: no symptoms

• Stage I: unimpaired ambulation; mostly mild sensory, motor, and autonomic neuropathy in the lower limbs

• Stage II: assistance with ambulation required; mostly moderate impairment progression to the lower limbs, upper limbs, and trunk

• Stage III: wheelchair-bound or bedridden; severe sensory, motor, and autonomic involvement of all limbs

#### PND: disease staging

• Stage 0: no impairment

• Stage I: sensory disturbances but preserved walking capability

• Stage II: impaired walking capability but ability to walk without a stick or crutches

• Stage IIIA: walking only with the help of one stick or crutch

• Stage IIIB: walking with the help of two sticks or crutches

• Stage IV: confined to a wheelchair or bedridden

#### Portuguese classification system to evaluate the severity of TTR-FAP

• Level 0: asymptomatic

• Level 1: sensitive and/or dysautonomic symptoms without neurologic signs

• Level 2: sensitive and/or dysautonomic symptoms with neurologic signs (sensitive)

• Level 3: sensitive and/or dysautonomic symptoms with neurologic signs (sensitive or motor) in the lower limbs with independent walking

• Level 4: neurologic signs in the lower and upper limbs (sensitive or motor), walking without help

• Level 5: neurologic signs (sensitive or motor) in the lower and upper limbs, in wheelchair

• Level 6: confined to bed

#### Sensory impairment (note the most proximal location [1, 2, or 3] for each sensation that is reduced)

Lower limbs

• Coldness (1: toe, 2: leg, 3: thigh)

• Pinprick (1: toe, 2: leg, 3: thigh)

• Light touch (1: toe, 2: leg, 3: thigh)

Upper limbs

• Coldness (1: finger, 2: elbow, 3: shoulder)

• Pinprick (1: finger, 2: elbow, 3: shoulder)

• Light touch (1: finger, 2: wrist, 3: elbow)

Trunk and head

• Coldness (1: umbilical level, 2: clavicular level, 3: neck and face)

• Pinprick (1: umbilical level, 2: clavicular level, 3: neck and face)

#### Autonomic dysfunction

• Diarrhea (2: alternating constipation and diarrhea, 4: regular diarrhea, 6: severe diarrhea)

• Orthostatic hypotension (2: systolic blood pressure 0–20 mm Hg decrease, 4: >20 mm Hg decrease, 6: severe, with faintness)

• Urination (2: mild, 4: incomplete retention, 6: permanent incontinence or retention)

• Dry eye (0: negative, 3: positive)

• Dry mouth (0: negative, 3: positive)

#### Motor function (muscle weakness)

• Anterior tibial (0: normal, 2: good, 3: fair, 4: poor, 5: trace, 6: zero)

• Quadriceps (0: normal, 2: good, 4: fair, 6: poor to zero)

• Wrist flexion (0: normal, 2: good, 3: fair, 4: poor, 5: trace, 6: zero)

• Biceps (0: normal, 2: good, 4: fair, 6: poor to zero)

• Scoring of visceral organ impairment

○ Heart (4: AV block I, 8: AV block II or sick sinus syndrome, 12: complete AV block)

○ Kidney (proteinuria [4: positive, 8: nephrotic syndrome], 12: renal failure)

## Appendix D

### Clinical trials

#### Tafamidis

The efficacy and safety of tafamidis were evaluated in a phase 2/3, randomized, double-blind, placebo-controlled clinical trial (Fx-005) in which patients received oral tafamidis 20 mg once daily (n = 65) or placebo (n = 63) for 18 months [88]. Co-primary end points were NIS-LL response to treatment (<2 point increase from baseline) at 18 months and change from baseline to 18 months in QOL as measured by a modified Norfolk Quality of Life–Diabetic Neuropathy (total QOL; TQOL) score. The intent-to-treat (ITT) population included all randomized patients who received ≥1 dose of study medication and had ≥1 post-baseline assessment for each primary end point, or who discontinued due to death or liver transplant. In this ITT population, while statistical significance was not reached for either of these endpoints, patients treated with tafamidis overall experienced numerically less disease progression and less worsening of quality of life than did patients treated with placebo. In the pre-specified efficacy evaluable population analysis at 18 months, the results showed a significantly greater percentage of patients in the tafamidis group with no disease progression based on the NIS-LL responder analysis when compared with the placebo group, while the tafamidis group demonstrated significantly less worsening in quality of life than the placebo group.

Tafamidis appeared to be safe and well tolerated; treatment-related adverse events included diarrhea, upper abdominal pain, urinary tract infection, and vaginal infection. An open-label extension study (n = 86) was conducted to evaluate long-term safety and efficacy [89]. Slowing of disease progression was observed in patients treated for either 30 or 18 months with tafamidis (i.e. the tafamidis-tafamidis and placebo-tafamidis cohorts, respectively), although earlier initiation of treatment was associated with better outcomes, including less neurologic deterioration and preserved nutritional status and QOL. No new safety concerns (as compared with the pivotal study) were observed over 30 months, and no patients discontinued treatment due to adverse events.

Two additional phase 2, open-label safety and efficacy studies of oral tafamidis 20 mg once daily in patients with non-Val30Met TTR amyloidosis have been completed, with results expected to be published in late 2012. In the polyneuropathy study, 21 patients with non-Val30Met TTR-FAP received tafamidis for 12 months, with TTR stabilization at week 6 compared with baseline as the primary outcome measure. Secondary outcome measures included the incidence of treatment-emergent adverse events and change from baseline in NIS and NIS-LL, nerve conduction studies, mBMI, TQOL, and NT-proBNP and troponin I cardiac biomarkers. Data suggest that non-Val30Met patients treated with tafamidis experience slower neurologic progression (NIS-LL) and sustained quality of life (TQOL) and nutritional status (mBMI) as compared with the placebo-treated Val30Met patients in the pivotal trial [90]. In the infiltrative cardiomyopathy study, 35 patients with wild-type or Val122Ile TTR amyloidosis with cardiomyopathy also received tafamidis once daily for 12 months, with TTR stabilization at week 6 compared with baseline as the primary end point, and secondary outcome measures, including incidence of treatment-emergent adverse events and change from baseline in echocardiography, cardiac MRI, chest x-ray, and Holter monitoring parameters, NT-proBNP, troponin I and T cardiac biomarkers, 6-minute walk test (6MWT), and health-related QOL measures. Compared with non-randomized, historical controls, patients receiving tafamidis treatment experienced smaller changes from baseline in NT-proBNP and 6MWT measures and a lower incidence of cardiovascular hospitalization/death at 12 months, although the differences were statistically insignificant [91]. Although patients in both studies were older and more severely affected compared with the Val30Met patients in the blinded study, tafamidis was effective in achieving and maintaining TTR tetramer stabilization, and no new safety concerns related to drug therapy were observed.

#### Diflunisal

A phase 2/3, randomized, double-blind, placebo-controlled, multinational clinical trial is currently under way evaluating the use of diflunisal 250 mg twice daily for preventing the progression of lower-limb nerve damage in patients with TTR-FAP. The primary end point is the Neurologic Impairment Score+7 (NIS+7) measured at 12 and 24 months. Secondary end points include the Kumamoto neurologic scale score, echocardiographic signs of cardiomyopathy, mBMI, amyloid deposition, and QOL. The study is scheduled to be completed at the end of 2012, with results announced in early 2013. Preliminary data indicate that diflunisal is well tolerated in the study population. An open-label extension study based in Sweden to observe the long-term effects (over the course of about 2 years) of diflunisal on neurologic and cardiac deterioration and nutritional status is currently recruiting patients. An open-label study in a single center in Japan (Shinshu University) reported significant stabilization of disease course [92].

#### ALN-TTR01 and ALN-TTR02

A phase 1, randomized, placebo-controlled, single-dose escalation study of ALN-TTR01 in up to 36 patients with TTR amyloidosis is near completion with results expected later in 2012. Preliminary results from a phase 1 trial of ALN-TTR02 indicate an up to 94% reduction of serum TTR levels and nearly 80% suppression after one month following a single dose, which was safe and well tolerated. An open-label phase 2 trial is currently underway.

#### Doxycycline/TUDCA

A 12-month, phase 2, open-label study to evaluate the pharmacokinetics, efficacy, safety, and tolerability of combined doxycycline/TUDCA treatment for TTR amyloidosis is ongoing. Twenty patients with both variant and wild-type TTR were enrolled, including three who had undergone liver transplants 8 to 15 years before initiation of the study. The primary end point was response rate to treatment (non-progression of neuropathy or cardiomyopathy), with responders defined as patients with mBMI reductions of <10% and NIS-LL changes of <2 points (in patients with TTR-FAP) or NT-proBNP concentration increases of <30% or <300 pg mL^–1^ (in patients with isolated cardiomyopathy). Secondary outcomes included pharmacokinetics, treatment-related adverse events, and change in QOL and neurologic assessments. Preliminary data suggest that the combined treatment can stabilize the disease and has an acceptable toxicity profile.

## Competing interests

YA, GS, FS, SI, WDL have no conflicts of interest to disclose.

JLB has received research support from Alnylam Pharmaceuticals and honoraria from Alnylam Pharmaceuticals, Isis Pharmaceuticals, Inc., and Pfizer Inc for consultation work.

TC’s institution received support from FoldRx Pharmaceuticals, which was acquired by Pfizer Inc in October 2010; she has served on the scientific advisory board of Pfizer Inc but was not compensated for this involvement, and received funding from Pfizer Inc for scientific meeting expenses (travel, accommodations, and registration) and for scientific lectures. She currently serves on the THAOS (natural history disease registry) scientific advisory board but does not receive compensation for this involvement.

MWC received support from FoldRx Pharmaceuticals, which was acquired by Pfizer Inc in October 2010, as a clinical investigator, has served on the scientific advisory board of Pfizer Inc, and received funding from Pfizer Inc for scientific meeting expenses (travel, accommodations, and registration). She currently serves on the THAOS (natural history disease registry) scientific advisory board but does not receive compensation for this involvement.

B-GE has received support from Pfizer Inc for scientific lectures.

LO has received support from Pfizer Inc as a consultant.

VP-B received support from FoldRx Pharmaceuticals, which was acquired by Pfizer Inc in October 2010, as a clinical investigator, and serves on the THAOS (natural history disease registry) scientific advisory board, but does not receive compensation for this involvement.

CR has received a single unrestricted research grant from Pfizer Inc.

## Authors’ contributions

All authors contributed to the initial concept for this manuscript, participated in drafting and revising the manuscript, and approved the final version for submission.

## References

[B1] Andrade C A peculiar form of peripheral neuropathy; familiar atypical generalized amyloidosis with special involvement of the peripheral nerves Brain 1952 75 408 427 10.1093/brain/75.3.408 12978172

[B2] Mutations in Hereditary Amyloidosis - Mutations in Transthyretin Gene (TTR) http://amyloidosismutations.com/mut-attr.php

[B3] Benson MD Kincaid JC The molecular biology and clinical features of amyloid neuropathy Muscle Nerve 2007 36 411 423 10.1002/mus.20821 17554795

[B4] Rapezzi C Quarta CC Obici L Perfetto F Longhi S Salvi F Biagini E Lorenzini M Grigioni F Leone O Disease profile and differential diagnosis of hereditary transthyretin-related amyloidosis with exclusively cardiac phenotype: an Italian perspective Eur Heart J 2012 Epub ahead of print 10.1093/eurheartj/ehs123 22745357

[B5] Harats N Worth RM Benson MD Hereditary amyloidosis: evidence against early amyloid deposition Arthritis Rheum 1989 32 1474 1476 10.1002/anr.1780321119 2510740

[B6] Hellman U Alarcon F Lundgren HE Suhr OB Bonaiti-Pellie C Plante-Bordeneuve V Heterogeneity of penetrance in familial amyloid polyneuropathy, ATTR Val30Met, in the Swedish population Amyloid 2008 15 181 186 10.1080/13506120802193720 18925456 PMC2738945

[B7] Planté-Bordeneuve V Carayol J Ferreira A Adams D Clerget-Darpoux F Misrahi M Said G Bonaiti-Pellie C Genetic study of transthyretin amyloid neuropathies: carrier risks among French and Portuguese families J Med Genet 2003 40 e120 10.1136/jmg.40.11.e120 14627687 PMC1735318

[B8] Saporta MA Zaros C Cruz MW Andre C Misrahi M Bonaiti-Pellie C Plante-Bordeneuve V Penetrance estimation of TTR familial amyloid polyneuropathy (type I) in Brazilian families Eur J Neurol 2009 16 337 341 10.1111/j.1468-1331.2008.02429.x 19364362

[B9] Bonaiti B Olsson M Hellman U Suhr O Bonaiti-Pellie C Plante-Bordeneuve V TTR familial amyloid polyneuropathy: does a mitochondrial polymorphism entirely explain the parent-of-origin difference in penetrance? Eur J Hum Genet 2010 18 948 952 10.1038/ejhg.2010.36 20234390 PMC2987385

[B10] Drugge U Andersson R Chizari F Danielsson M Holmgren G Sandgren O Sousa A Familial amyloidotic polyneuropathy in Sweden: a pedigree analysis J Med Genet 1993 30 388 392 10.1136/jmg.30.5.388 8100581 PMC1016374

[B11] Sousa A Coelho T Barros J Sequeiros J Genetic epidemiology of familial amyloidotic polyneuropathy (FAP)-type I in Povoa do Varzim and Vila do Conde (north of Portugal) Am J Med Genet 1995 60 512 521 10.1002/ajmg.1320600606 8825887

[B12] Yamamoto K Ikeda S Hanyu N Takeda S Yanagisawa N A pedigree analysis with minimised ascertainment bias shows anticipation in Met30-transthyretin related familial amyloid polyneuropathy J Med Genet 1998 35 23 30 10.1136/jmg.35.1.23 9475090 PMC1051182

[B13] Ohmori H Ando Y Makita Y Onouchi Y Nakajima T Saraiva MJ Terazaki H Suhr O Sobue G Nakamura M Common origin of the Val30Met mutation responsible for the amyloidogenic transthyretin type of familial amyloidotic polyneuropathy J Med Genet 2004 41 e51 10.1136/jmg.2003.014803 15060127 PMC1735751

[B14] Soares ML Coelho T Sousa A Holmgren G Saraiva MJ Kastner DL Buxbaum JN Haplotypes and DNA sequence variation within and surrounding the transthyretin gene: genotype-phenotype correlations in familial amyloid polyneuropathy (V30M) in Portugal and Sweden Eur J Hum Genet 2004 12 225 237 10.1038/sj.ejhg.5201095 14673473

[B15] Zaros C Genin E Hellman U Saporta MA Languille L Wadington-Cruz M Suhr O Misrahi M Plante-Bordeneuve V On the origin of the transthyretin Val30Met familial amyloid polyneuropathy Ann Hum Genet 2008 72 478 484 10.1111/j.1469-1809.2008.00439.x 18460047

[B16] Conceição I De Carvalho M Clinical variability in type I familial amyloid polyneuropathy (Val30Met): comparison between late- and early-onset cases in Portugal Muscle Nerve 2007 35 116 118 10.1002/mus.20644 16969832

[B17] Benson M Scriver CR, Beaudet AL, Valle D, Sly WS, Childs B, Kinzler KW, Vogelstein B Amyloidosis The Metabolic and Molecular Bases of Inherited Disease 2000 New York: McGraw-Hill 5345 5378

[B18] Prevalence of rare diseases: Bibliographic data (November 2011) http://www.orpha.net/orphacom/cahiers/docs/GB/Prevalence_of_rare_diseases_by_alphabetical_list.pdf

[B19] Kato-Motozaki Y Ono K Shima K Morinaga A Machiya T Nozaki I Shibata-Hamaguchi A Furukawa Y Yanase D Ishida C Epidemiology of familial amyloid polyneuropathy in Japan: Identification of a novel endemic focus J Neurol Sci 2008 270 133 140 10.1016/j.jns.2008.02.019 18410945

[B20] Jacobson DR Pastore R Pool S Malendowicz S Kane I Shivji A Embury SH Ballas SK Buxbaum JN Revised transthyretin Ile 122 allele frequency in African-Americans Hum Genet 1996 98 236 238 10.1007/s004390050199 8698351

[B21] Yamashita T Hamidi Asl K Yazaki M Benson MD A prospective evaluation of the transthyretin Ile122 allele frequency in an African-American population Amyloid 2005 12 127 130 10.1080/13506120500107162 16011990

[B22] Buxbaum J Alexander A Koziol J Tagoe C Fox E Kitzman D Significance of the amyloidogenic transthyretin Val 122 Ile allele in African Americans in the Arteriosclerosis Risk in Communities (ARIC) and Cardiovascular Health (CHS) Studies Am Heart J 2010 159 864 870 10.1016/j.ahj.2010.02.006 20435197 PMC2865207

[B23] Dharmarajan K Maurer MS Transthyretin Cardiac Amyloidoses in Older North Americans J Am Geriatr Soc 2012 60 765 774 10.1111/j.1532-5415.2011.03868.x 22329529 PMC3325376

[B24] Ikeda S Nakazato M Ando Y Sobue G Familial transthyretin-type amyloid polyneuropathy in Japan: clinical and genetic heterogeneity Neurology 2002 58 1001 1007 10.1212/WNL.58.7.1001 11940682

[B25] Sousa A Andersson R Drugge U Holmgren G Sandgren O Familial amyloidotic polyneuropathy in Sweden: geographical distribution, age of onset, and prevalence Hum Hered 1993 43 288 294 10.1159/000154146 8406517

[B26] Holmgren G Costa PM Andersson C Asplund K Steen L Beckman L Nylander PO Teixeira A Saraiva MJ Costa PP Geographical distribution of TTR met30 carriers in northern Sweden: discrepancy between carrier frequency and prevalence rate J Med Genet 1994 31 351 354 10.1136/jmg.31.5.351 8064809 PMC1049863

[B27] Misu K Hattori N Nagamatsu M Ikeda S Ando Y Nakazato M Takei Y Hanyu N Usui Y Tanaka F Late-onset familial amyloid polyneuropathy type I (transthyretin Met30-associated familial amyloid polyneuropathy) unrelated to endemic focus in Japan. Clinicopathological and genetic features Brain 1999 122 Pt 10 1951 1962 10506096 10.1093/brain/122.10.1951

[B28] Ando Y Araki S Ando M Transthyretin and familial amyloidotic polyneuropathy Intern Med 1993 32 920 922 10.2169/internalmedicine.32.920 8204970

[B29] Ando Y Suhr OB Autonomic dysfunction in familial amyloidotic polyneuropathy (FAP) Amyloid 1998 5 288 300 10.3109/13506129809007303 10036588

[B30] Koike H Misu K Ikeda S Ando Y Nakazato M Ando E Yamamoto M Hattori N Sobue G Type I (transthyretin Met30) familial amyloid polyneuropathy in Japan: early- vs late-onset form Arch Neurol 2002 59 1771 1776 10.1001/archneur.59.11.1771 12433265

[B31] Koike H Misu K Sugiura M Iijima M Mori K Yamamoto M Hattori N Mukai E Ando Y Ikeda S Sobue G Pathology of early- vs late-onset TTR Met30 familial amyloid polyneuropathy Neurology 2004 63 129 138 10.1212/01.WNL.0000132966.36437.12 15249622

[B32] Koike H Tanaka F Hashimoto R Tomita M Kawagashima Y Iima M Fujitake J Kawanami T Kato T Yamamoto M Sobue G Natural history of transthyretin Val30Met familial amyloid polyneuropathy: analysis of late-onset cases from non-endemic areas J Neurol Neurosurg Psychiatry 2012 83 152 158 10.1136/jnnp-2011-301299 22228785

[B33] Koike H Ando Y Ueda M Kawagashira Y Iijima M Fujitake J Hayashi M Yamamoto M Mukai E Nakamura T Distinct characteristics of amyloid deposits in early- and late-onset transthyretin Val30Met familial amyloid polyneuropathy J Neurol Sci 2009 287 178 184 10.1016/j.jns.2009.07.028 19709674

[B34] Wang AK Fealey RD Gehrking TL Low PA Patterns of neuropathy and autonomic failure in patients with amyloidosis Mayo Clin Proc 2008 83 1226 1230 10.4065/83.11.1226 18990321 PMC2842947

[B35] Benson M Richardson SJ, Cody V Genetics: Clinical Implications of TTR Amyloidosis Recent Advances in Transthyretin Evolution, Structure and Biological Functions 2009 Berlin: Springer 173 189

[B36] Beirao I Lobato L Costa PM Fonseca I Mendes P Silva M Bravo F Cabrita A Porto G Kidney and anemia in familial amyloidosis type I Kidney Int 2004 66 2004 2009 10.1111/j.1523-1755.2004.00971.x 15496172

[B37] Ando E Ando Y Okamura R Uchino M Ando M Negi A Ocular manifestations of familial amyloidotic polyneuropathy type I: long-term follow up Br J Ophthalmol 1997 81 295 298 10.1136/bjo.81.4.295 9215058 PMC1722164

[B38] Falk RH Dubrey SW Amyloid heart disease Prog Cardiovasc Dis 2010 52 347 361 10.1016/j.pcad.2009.11.007 20109604

[B39] Shah KB Inoue Y Mehra MR Amyloidosis and the heart: a comprehensive review Arch Intern Med 2006 166 1805 1813 10.1001/archinte.166.17.1805 17000935

[B40] Planté-Bordeneuve V Ferreira A Lalu T Zaros C Lacroix C Adams D Said G Diagnostic pitfalls in sporadic transthyretin familial amyloid polyneuropathy (TTR-FAP) Neurology 2007 69 693 698 10.1212/01.wnl.0000267338.45673.f4 17698792

[B41] Rapezzi C Perugini E Salvi F Grigioni F Riva L Cooke RM Ferlini A Rimessi P Bacchi-Reggiani L Ciliberti P Phenotypic and genotypic heterogeneity in transthyretin-related cardiac amyloidosis: towards tailoring of therapeutic strategies? Amyloid 2006 13 143 153 10.1080/13506120600877136 17062380

[B42] Rapezzi C Quarta CC Riva L Longhi S Gallelli I Lorenzini M Ciliberti P Biagini E Salvi F Branzi A Transthyretin-related amyloidoses and the heart: a clinical overview Nat Rev Cardiol 2010 7 398 408 10.1038/nrcardio.2010.67 20479782

[B43] Ikeda S Cardiac amyloidosis: heterogenous pathogenic backgrounds Intern Med 2004 43 1107 1114 10.2169/internalmedicine.43.1107 15645642

[B44] Elliott P Andersson B Arbustini E Bilinska Z Cecchi F Charron P Dubourg O Kuhl U Maisch B McKenna WJ Classification of the cardiomyopathies: a position statement from the European Society of Cardiology Working Group on Myocardial and Pericardial Diseases Eur Heart J 2008 29 270 276 17916581 10.1093/eurheartj/ehm342

[B45] Koyama J Ray-Sequin PA Davidoff R Falk RH Usefulness of pulsed tissue Doppler imaging for evaluating systolic and diastolic left ventricular function in patients with AL (primary) amyloidosis Am J Cardiol 2002 89 1067 1071 10.1016/S0002-9149(02)02277-4 11988197

[B46] Koyama J Davidoff R Falk RH Longitudinal myocardial velocity gradient derived from pulsed Doppler tissue imaging in AL amyloidosis: a sensitive indicator of systolic and diastolic dysfunction J Am Soc Echocardiogr 2004 17 36 44 10.1016/j.echo.2003.09.014 14712185

[B47] Rapezzi C Quarta CC Guidalotti PL Longhi S Pettinato C Leone O Ferlini A Salvi F Gallo P Gagliardi C Branzi A Usefulness and limitations of 99mTc-3,3-diphosphono-1,2-propanodicarboxylic acid scintigraphy in the aetiological diagnosis of amyloidotic cardiomyopathy Eur J Nucl Med Mol Imaging 2011 38 470 478 10.1007/s00259-010-1642-7 21069320

[B48] Quarta CC Guidalotti PL Longhi S Pettinato C Leone O Ferlini A Biagini E Grigioni F Bacchi-Reggiani ML Lorenzini M Defining the diagnosis in echocardiographically suspected senile systemic amyloidosis JACC Cardiovasc Imaging 2012 5 755 758 10.1016/j.jcmg.2012.02.015 22789945

[B49] Mitsuhashi S Yazaki M Tokuda T Sekijima Y Washimi Y Shimizu Y Ando Y Benson MD Ikeda S Biochemical characteristics of variant transthyretins causing hereditary leptomeningeal amyloidosis Amyloid 2005 12 216 225 10.1080/13506120500352404 16399646

[B50] Suhr O Danielsson A Holmgren G Steen L Malnutrition and gastrointestinal dysfunction as prognostic factors for survival in familial amyloidotic polyneuropathy J Intern Med 1994 235 479 485 10.1111/j.1365-2796.1994.tb01106.x 8182405

[B51] Vital C Vital A Bouillot-Eimer S Brechenmacher C Ferrer X Lagueny A Amyloid neuropathy: a retrospective study of 35 peripheral nerve biopsies J Peripher Nerv Syst 2004 9 232 241 10.1111/j.1085-9489.2004.09405.x 15574136

[B52] Guy CD Jones CK Abdominal fat pad aspiration biopsy for tissue confirmation of systemic amyloidosis: Specificity, positive predictive value, and diagnostic pitfalls Diagn Cytopathol 2001 24 181 185 10.1002/1097-0339(200103)24:3<181::AID-DC1037>3.0.CO;2-D 11241901

[B53] Kaplan B Vidal R Kumar A Ghiso J Gallo G Immunochemical microanalysis of amyloid proteins in fine-needle aspirates of abdominal fat Am J Clin Pathol 1999 112 403 407 10478147 10.1093/ajcp/112.3.403

[B54] Tsuchiya A Yazaki M Kametani F Takei Y Ikeda S Marked regression of abdominal fat amyloid in patients with familial amyloid polyneuropathy during long-term follow-up after liver transplantation Liver Transpl 2008 14 563 570 10.1002/lt.21395 18383093

[B55] Coutinho P Martins da Silva A Lopes Lima J Resende Barbosa A Glenner GG, Pinho e Costa P, Falcao de Freitas A Forty years of experience with type I amyloid neuropathy. Review of 483 cases Amyloid and amyloidosis 1980 Amsterdam: Excerpta Medica 88 98

[B56] Sales-Luís ML Galvão M Carvalho M Sousa G Alves MM Serrão R Treatment of familial amyloidotic polyneuropathy (Portuguese type) by plasma exchange Muscle Nerve 1991 14 377 378 2027354

[B57] Herlenius G Wilczek HE Larsson M Ericzon BG Ten years of international experience with liver transplantation for familial amyloidotic polyneuropathy: results from the Familial Amyloidotic Polyneuropathy World Transplant Registry Transplantation 2004 77 64 71 10.1097/01.TP.0000092307.98347.CB 14724437

[B58] Ihse E Suhr OB Hellman U Westermark P Variation in amount of wild-type transthyretin in different fibril and tissue types in ATTR amyloidosis J Mol Med (Berl) 2011 89 171 180 10.1007/s00109-010-0695-1 21107516 PMC3022153

[B59] Wilczek HE Larsson M Ericzon BG Long-term data from the Familial Amyloidotic Polyneuropathy World Transplant Registry (FAPWTR) Amyloid 2011 18 Suppl 1 188 190 10.3109/13506129.2011.574354072 21838484

[B60] Stangou AJ Heaton ND Hawkins PN Transmission of systemic transthyretin amyloidosis by means of domino liver transplantation N Engl J Med 2005 352 2356 10.1056/NEJM200506023522219 15930432

[B61] Sousa MM Ferrão J Fernandes R Guimaraes A Geraldes JB Perdigoto R Tome L Mota O Negrao L Furtado AL Saraiva MJ Deposition and passage of transthyretin through the blood-nerve barrier in recipients of familial amyloid polyneuropathy livers Lab Invest 2004 84 865 873 10.1038/labinvest.3700107 15122304

[B62] Lladó L Baliellas C Casasnovas C Ferrer I Fabregat J Ramos E Castellote J Torras J Xiol X Rafecas A Risk of transmission of systemic transthyretin amyloidosis after domino liver transplantation Liver Transpl 2010 16 1386 1392 10.1002/lt.22174 21117248

[B63] Arpesella G Chiappini B Marinelli G Mikus PM Dozza F Pierangeli A Magelli C Salvi F Leone O Combined heart and liver transplantation for familial amyloidotic polyneuropathy J Thorac Cardiovasc Surg 2003 125 1165 1166 10.1067/mtc.2003.151 12771895

[B64] Said G Grippon S Kirkpatrick P Tafamidis Nat Rev Drug Discov 2012 11 185 186 10.1038/nrd3675 22378262

[B65] Berk JL Suhr OB Sekijima Y Yamashita T Heneghan M Zeldenrust SR Ando Y Ikeda S Gorevic P Merlini G The Diflunisal Trial: study accrual and drug tolerance Amyloid 2012 19 Suppl 1 37 38 22551208 10.3109/13506129.2012.678509

[B66] Ackermann EJ Guo S Booten S Alvarado L Benson M Hughes S Monia BP Clinical development of an antisense therapy for the treatment of transthyretin-associated polyneuropathy Amyloid 2012 19 Suppl 1 43 44 22494066 10.3109/13506129.2012.673140

[B67] Cardoso I Martins D Ribeiro T Merlini G Saraiva MJ Synergy of combined doxycycline/TUDCA treatment in lowering transthyretin deposition and associated biomarkers: studies in FAP mouse models J Transl Med 2010 8 74 10.1186/1479-5876-8-74 20673327 PMC2922089

[B68] Obici L Cortese A Lozza A Lucchetti J Gobbi M Palladini G Perlini S Saraiva MJ Merlini G Doxycycline plus tauroursodeoxycholic acid for transthyretin amyloidosis: a phase II study Amyloid 2012 19 Suppl 1 34 36 22551192 10.3109/13506129.2012.678508

[B69] Gertz MA Comenzo R Falk RH Fermand JP Hazenberg BP Hawkins PN Merlini G Moreau P Ronco P Sanchorawala V Definition of organ involvement and treatment response in immunoglobulin light chain amyloidosis (AL): a consensus opinion from the 10th International Symposium on Amyloid and Amyloidosis, Tours, France, 18–22 April 2004 Am J Hematol 2005 79 319 328 10.1002/ajh.20381 16044444

[B70] Rapezzi C Merlini G Quarta CC Riva L Longhi S Leone O Salvi F Ciliberti P Pastorelli F Biagini E Systemic cardiac amyloidoses: disease profiles and clinical courses of the 3 main types Circulation 2009 120 1203 1212 10.1161/CIRCULATIONAHA.108.843334 19752327

[B71] Sun JP Stewart WJ Yang XS Donnell RO Leon AR Felner JM Thomas JD Merlino JD Differentiation of hypertrophic cardiomyopathy and cardiac amyloidosis from other causes of ventricular wall thickening by two-dimensional strain imaging echocardiography Am J Cardiol 2009 103 411 415 10.1016/j.amjcard.2008.09.102 19166699

[B72] Selvanayagam JB Hawkins PN Paul B Myerson SG Neubauer S Evaluation and management of the cardiac amyloidosis J Am Coll Cardiol 2007 50 2101 2110 10.1016/j.jacc.2007.08.028 18036445

[B73] Perugini E Rapezzi C Reggiani LB Poole-Wilson P Branzi A Henein MY Comparison of ventricular long-axis function in patients with cardiac amyloidosis versus idiopathic restrictive cardiomyopathy Am J Cardiol 2005 95 146 149 10.1016/j.amjcard.2004.08.084 15619416

[B74] Rahman JE Helou EF Gelzer-Bell R Thompson RE Kuo C Rodriguez ER Hare JM Baughman KL Kasper EK Noninvasive diagnosis of biopsy-proven cardiac amyloidosis J Am Coll Cardiol 2004 43 410 415 10.1016/j.jacc.2003.08.043 15013123

[B75] Murtagh B Hammill SC Gertz MA Kyle RA Tajik AJ Grogan M Electrocardiographic findings in primary systemic amyloidosis and biopsy-proven cardiac involvement Am J Cardiol 2005 95 535 537 10.1016/j.amjcard.2004.10.028 15695149

[B76] Maceira AM Joshi J Prasad SK Moon JC Perugini E Harding I Sheppard MN Poole-Wilson PA Hawkins PN Pennell DJ Cardiovascular magnetic resonance in cardiac amyloidosis Circulation 2005 111 186 193 10.1161/01.CIR.0000152819.97857.9D 15630027

[B77] Perugini E Rapezzi C Piva T Leone O Bacchi-Reggiani L Riva L Salvi F Lovato L Branzi A Fattori R Non-invasive evaluation of the myocardial substrate of cardiac amyloidosis by gadolinium cardiac magnetic resonance Heart 2006 92 343 349 15939726 10.1136/hrt.2005.061911 PMC1860803

[B78] Glaudemans AW Slart RH Zeebregts CJ Veltman NC Tio RA Hazenberg BP Dierckx RA Nuclear imaging in cardiac amyloidosis Eur J Nucl Med Mol Imaging 2009 36 702 714 10.1007/s00259-008-1037-1 19156411

[B79] Hazenberg BP van Rijswijk MH Lub-de Hooge MN Vellenga E Haagsma EB Posthumus MD Jager PL Diagnostic performance and prognostic value of extravascular retention of 123I-labeled serum amyloid P component in systemic amyloidosis J Nucl Med 2007 48 865 872 10.2967/jnumed.106.039313 17504868

[B80] Hawkins PN Serum amyloid P component scintigraphy for diagnosis and monitoring amyloidosis Curr Opin Nephrol Hypertens 2002 11 649 655 10.1097/00041552-200211000-00013 12394612

[B81] Hawkins PN Lavender JP Pepys MB Evaluation of systemic amyloidosis by scintigraphy with 123I-labeled serum amyloid P component N Engl J Med 1990 323 508 513 10.1056/NEJM199008233230803 2377176

[B82] Aprile C Marinone G Saponaro R Bonino C Merlini G Cardiac and pleuropulmonary AL amyloid imaging with technetium-99m labelled aprotinin Eur J Nucl Med 1995 22 1393 1401 10.1007/BF01791147 8586084

[B83] Han S Chong V Murray T McDonagh T Hunter J Poon FW Gray HW Neilly JB Preliminary experience of 99mTc-Aprotinin scintigraphy in amyloidosis Eur J Haematol 2007 79 494 500 10.1111/j.1600-0609.2007.00963.x 17983443

[B84] Schaadt BK Hendel HW Gimsing P Jonsson V Pedersen H Hesse B 99mTc-aprotinin scintigraphy in amyloidosis J Nucl Med 2003 44 177 183 12571206

[B85] Hongo M Urushibata K Kai R Takahashi W Koizumi T Uchikawa S Imamura H Kinoshita O Owa M Fujii T Iodine-123 metaiodobenzylguanidine scintigraphic analysis of myocardial sympathetic innervation in patients with AL (primary) amyloidosis Am Heart J 2002 144 122 129 10.1067/mhj.2002.123115 12094198

[B86] Tanaka M Hongo M Kinoshita O Takabayashi Y Fujii T Yazaki Y Isobe M Sekiguchi M Iodine-123 metaiodobenzylguanidine scintigraphic assessment of myocardial sympathetic innervation in patients with familial amyloid polyneuropathy J Am Coll Cardiol 1997 29 168 174 10.1016/S0735-1097(96)00438-X 8996310

[B87] Perugini E Guidalotti PL Salvi F Cooke RM Pettinato C Riva L Leone O Farsad M Ciliberti P Bacchi-Reggiani L Noninvasive etiologic diagnosis of cardiac amyloidosis using 99mTc-3,3-diphosphono-1,2-propanodicarboxylic acid scintigraphy J Am Coll Cardiol 2005 46 1076 1084 10.1016/j.jacc.2005.05.073 16168294

[B88] Coelho T Maia LF Martins da Silva A Waddington Cruz M Plante-Bordeneuve V Lozeron P Suhr OB Campistol JM Conceicao IM Schmidt HH Tafamidis for transthyretin familial amyloid polyneuropathy: a randomized, controlled trial Neurology 2012 79 785 792 10.1212/WNL.0b013e3182661eb1 22843282 PMC4098875

[B89] Coelho T New pharmacological treatment J Neurol 2011 258 S4

[B90] Planté-Bordeneuve V Schmidt H Merlini G Judge DP Obici L Packman J Grogan DR The effects of tafamidis on transthyretin stabilization and clinical outcomes in patients with non-V30M transthyretin amyloidosis Eur J Neurol 2011 18 S29 10.1007/s12265-013-9512-x PMC3838581 24101373

[B91] Falk RH Maurer MS Fedson SE Judge DP Zeldenrust SR Quyyumi A Pano A Packman J Grogan DR Tafamidis stabilizes transthyretin and improves clinical outcomes in transthyretin amyloid cardiomyopathy J Cardiac Fail 2011 17 S56

[B92] Sekijima Y Familial amyloid polyneuropathy: diflunisal Rinsho Shinkeigaku 2010 50 836 10.5692/clinicalneurol.50.836 21921462

